# An experimental approach: Investigating the directive function of autobiographical memory

**DOI:** 10.3758/s13421-023-01480-w

**Published:** 2023-10-24

**Authors:** Nicky Duff, Karen Salmon, Anne Macaskill

**Affiliations:** School of Psychology, Victoria University of Wellington, PO Box 600, Wellington, New Zealand

**Keywords:** Autobiographical memory, Directive function, Open-ended problem solving, Episodic detail, Self-efficacy

## Abstract

Why do we have autobiographical memory and how is it useful? Researchers have proposed a directive function; our experiences guide our behavior, particularly when faced with an open-ended problem. Two experiments (one between-participant and one mixed design) were therefore conducted to test whether success autobiographical memories – any experience when the participant felt successful and competent – are helpful for generating solutions to problem scenarios. One research aim was to experimentally test the directive function as current experimental evidence is limited and results are mixed. Consequently, it is unclear if and how autobiographical memory is helpful for open-ended problem solving. Another aim was to test whether self-efficacy is an important factor that supports open-ended problem solving and thus the directive function. Although success memories enhanced self-ratings of self-efficacy across both experiments, in samples of undergraduate students there was no experimental effect of success autobiographical memories on problem solving. Instead, some participants across the memory and control conditions in both experiments, even when not instructed, recalled autobiographical memories related to the problem scenarios presented in the problem-solving task, and these participants did better at problem solving than those who did not. This may hint to a directive function and is perhaps one reason why there is no experimental effect. Sample and experimental design differences are discussed as potential factors that may contribute to non-significant effects in this study but significant effects in others. Our results highlight the complexity of the directive function, and the difficulty of experimentally testing how autobiographical memory directs behavior.

## Introduction

Autobiographical memory is our memory of personal events, and our knowledge and subjective experience of the self across time (Conway, 2005; Tulving, 2002). But why do we have autobiographical memory? How is it useful? Researchers have proposed that autobiographical memories serve a variety of adaptive functions, such as a self (i.e., promotes the development and continuity of the self across time), a social (i.e., develops, maintains, and enhances relationships), and a directive (i.e., guides and directs behavior during problems) function (Bluck 2003; Bluck et al., 2005; Bluck et al, 2019; Pillemer, 1992; Pillemer & Kuwabara, 2012). The directive function has received less research attention than the other two (Bluck, 2003; Pillemer, 2003), particularly with respect to experimental research (Beike et al., 2020; Pillemer, 2009; Pillemer & Kuwabara, 2012). It is therefore unclear whether and how autobiographical memory directs and guides behavior during problems. Taking an experimental approach, the focus of this study was to investigate the conditions under which autobiographical memories direct behavior during problem solving.

## What is the ‘Directive Function of Autobiographical Memory’?

From a theoretical perspective, autobiographical memories serve to inform, guide, motivate, and inspire (Pillemer, 2003). Indeed, how people construct their life story has been shown to influence the goals they pursue, the values they set, and their wellbeing (Fivush, 2011; McAdams & Manczak, 2015). Thus, the directive function definition can often be broad, such that autobiographical memory can influence many thoughts, feelings, and behavior (e.g., Bluck & Alea, 2002; Bluck et al., 2019; Pillemer & Kuwabara, 2012). Researchers converge on the idea that autobiographical memories direct and guide behavior during problem solving, however; because they provide information and lessons that can be applied to current or future problems (Bluck & Alea, 2009, 2011; Harris et al., 2014; Kuwabara & Pillemer, 2010). Autobiographical memories are expected to be particularly useful during open-ended problem solving where multiple solutions are possible and there is no one standard solution (Bluck & Alea, 2009; Olivares, 2010; Pillemer, 2001). That is, a person draws on the content of an autobiographical memory to help them navigate their open-ended problem.

The definition of and evidence for the directive function has come predominately from self-report questionnaires. The Thinking About Life Experiences Questionnaire (TALE; Bluck, et al., 2005; or the revised version TALE-R; Bluck & Alea, 2011) and the Reminiscence Functions Scale (RFS; Webster, 1993, 1997, 2003) are prominent scales, with many papers referencing their findings as evidence for autobiographical memory functions. The TALE and RFS ask people how they use their autobiographical memory in their daily lives. Factor analyses of the TALE and RFS responses reveal a ‘directive’ factor and ’problem-solving’ factor (i.e., how remembering past problem-solving strategies helps with present problems), respectively, with both having high factor loadings and good internal consistency (Bluck et al, 2005, Bluck & Alea, 2011; Webster, 1993, 1997, 2003). The ‘directive’ and ‘problem solving’ factors also highly correlate with each other, providing convergent validity for the directive function of autobiographical memory (Bluck et al., 2005). Thus, TALE and RFS findings show that people report that their autobiographical memory guides their current and future behavior during problem solving, particularly when they are uncertain about which path to take.

Surveys and factor analysis are a helpful starting point to understand the ways in which autobiographical memory recall benefits a person, but people may not have insight into whether, and how, autobiographical memory recall is beneficial (Beike et al., 2020; Bluck & Alea, 2011; Hyman & Faries, 1992; Pillemer & Kuwabara, 2012; Sow et al., 2022). Additionally, although the TALE and RFS provide correlational evidence for a directive function, this type of evidence does not show whether autobiographical memory recall causes better problem solving. Experimental evidence will therefore help to clarify whether and how recalling autobiographical memories can influence open-ended problem solving.

## Experimental evidence for the directive function: The role of self-efficacy

Experimental evidence for the directive function is limited and mixed, however, with only two papers using the directive function theory and research as rationale for their experiment (i.e., Biondolillo & Pillemer, 2015; Kuwabara & Pillemer, 2010). Therefore, we drew on research that found an effect of autobiographical memory on problem solving or helping a person overcome a challenge (Biondolillo & Pillemer, 2015; Brown et al., 2016; Raeder et al., 2019; Pezdek & Salim, 2011). A key factor that appeared in these studies was self-efficacy, which is defined as the belief or the confidence one holds that they can perform a specific behavior and achieve certain goals (Bandura, 1997). That is, drawing a sense of self-efficacy from a memory about successfully overcoming a problem can help with the current problem or challenge (Biondolillo & Pillemer, 2015; Brown et al., 2016; Raeder et al., 2019; Pezdek & Salim, 2011).

For instance, compared to a no-recall control condition, undergraduate students who recalled a detailed and specific experience where they were pleased and satisfied during or after exercising, increased their exercise over a week (Biondolillo & Pillemer, 2015). This effect held even after controlling for prior attitudes, motivation, and exercise activity. Furthermore, evaluating an experience of successfully overcoming a fear of heights, in addition to a virtual reality height exposure program, helped people overcome acrophobia (Raeder et al., 2019). That is, only participants who recalled a success experience and reflected on how they overcame their fear (compared to those that had no recall or evaluation prompt) significantly increased their self-reported self-efficacy (operationalized as feelings of self-confidence) and decreased their self-reported fear (Raeder et al., 2019). Those participants also decreased their avoidant behavior in an in vivo 24-h and 4-week post-treatment behavioral activation test (Raeder et al., 2019). Further, high school students who recalled a positive public speaking experience before public speaking demonstrated enhanced performance and lowered anxiety compared to students who recalled experiences of overcoming an animal or medical aversion (Pezdek & Salim, 2011).

Brown et al. (2016) found more direct evidence for self-efficacious autobiographical memories helping with open-ended, uncertain problems because problem solving in their study was measured by the Means-Ends Problem Solving task (MEPS; Platt & Spivack, 1975). The MEPS task asks participants to generate solutions, steps, or ideal strategies that the protagonist in a problem scenario (e.g., making friends, resolving a conflict) could take to reach a positive ending. The scenarios are open-ended problems that do not have one standard solution, and so the number of solutions that move the person in the problem scenario toward the positive ending is counted (or in some studies, the extent of the solutions’ effectiveness, e.g., Goddard et al., 1996, 1997, 2001). Brown and colleagues asked two groups of American combat veterans, one without and one with PTSD (post-traumatic stress disorder), to either recall three autobiographical memories of when they overcame a challenge and felt successful and competent (i.e., success-memory condition) or recall any three significant life events (i.e., control condition). In the success-memory condition, the researchers also discussed with the participant how their success experience reflected their strengths. Participants then completed the MEPS task, which had been adapted to incorporate open-ended problems particularly relevant to veterans (e.g., finding work after being discharged from the army). In the success-memory condition, participants’ self-reported levels of self-efficacy (operationalized as feelings of self-confidence) significantly increased, and they produced significantly more solutions than the control condition, regardless of PTSD status.

Interestingly, however, Beaman et al. (2007) found that recalling cued positive and negative autobiographical memories before the MEPS did not facilitate problem solving. That is, older (61–83 years) and younger (19–25 years) participants who recalled autobiographical memories, cued from positive or negative words (i.e., Autobiographical Memory Test, AMT; Williams & Broadbent, 1986) before the MEPS task did not generate significantly more solutions compared to older and younger participants who did the tasks in the reverse order. Similarly, Goddard et al. (2001) found that recalling autobiographical memories related to the problem scenario did not enhance problem solving. Depressed and non-depressed participants who recalled autobiographical memories that the problem scenarios reminded them of did not generate significantly more solutions compared to participants who were not asked to recall autobiographical memories (Goddard et al., 2001). Thus, cued autobiographical memory recall from positive and negative words, and recalling an experience that reminds one of the problem, did not improve subsequent open-ended problem solving as measured by the MEPS.

Considered together, the mixed findings of these experimental studies raise the possibility that whether autobiographical memories enhance open-ended problem solving may depend on how the memory elicits a person’s self-efficacy. That is, to help a person overcome a problem, autobiographical memory may need to enhance self-efficacy. Under some circumstances, the autobiographical memory could be *any* experience that makes a person feel competent and successful, not necessarily one related to the problem (Brown et al., 2016). Indeed, recalling a mastery experience from a particular domain of functioning may influence efficacy beliefs in other domains of functioning by providing a person compelling evidence of their ability (Bandura, 1997).

Self-efficacy is the confidence a person has in their ability to control their motivation, behavior, and environment (Bandura, 1997). Heightened self-efficacy can therefore have a subsequent effect on behavior, particularly during open-ended problem solving (Bandura, 1997; Brown et al., 2012; Brown et al., 2016; D’Zurilla et al., 2004). This may be because being optimistic and confident in one's ability to solve problems leads to a better and more effective open-ended problem-solving approach, including generating solutions to problems (D’Zurilla et al., 2004). Therefore, mastery autobiographical memories of when a person felt successful and competent may enhance self-efficacy, which subsequently motivates a person to solve the problem, because they believe they can and thus they put in effort and commitment to do so (Bandura, 1997; D’Zurilla et al., 2004).

## The relationship between the self and directive function

Although experimental evidence for the directive function suggests self-efficacy may be an important factor in directing behavior during problem solving, self-efficacy is not often discussed in the literature on the directive function. Yet interestingly the findings from the TALE, TALE-R, and RFS scales link the self and the directive functions together (Bluck et al., 2005; Bluck & Alea, 2011; Webster, 1993, 1997, 2003). To illustrate, an exploratory factor analysis of the TALE items found that an item about recalling an autobiographical memory to raise self-confidence during a challenge loaded onto the directive function factor and had good internal consistency with the other items focused on lessons learnt (Bluck et al., 2005). The initial development of the RFS also saw that the identity function (i.e., like the self function from the TALE/TALE-R; to discover and crystalize identity) and the problem-solving function merged as one “identity/problem solving” factor (Webster, 1993). Even when these factors were separated in subsequent papers by changing the factor rotation, the factors stayed strongly correlated (Webster, 1997, 2003). Harris et al. (2014) also looked at the relationship between the TALE-R questionnaire and RFS and found a “Reflective” factor that included the directive and self factors from the TALE-R questionnaire, and the identity and problem-solving factors from the RFS. Harris and colleagues therefore posited that it could be difficult to conceptually tease the self and directive functions apart, and that it may be more useful to consider them together.

From a theoretical perspective, the close relationship between the self and the directive functions makes sense as the self is intimately linked to autobiographical memory (Conway, 2005; Conway & Pleydell-Pearce, 2000; Prebble et al., 2013). A person’s experiences shape their self-concept which is the collection of ideas about oneself, like qualities, traits, roles and beliefs (e.g., I am a diligent researcher; Conway et al., 2004a, b; Prebble et al., 2013). A person’s self-concept therefore influences a person’s beliefs about the self (e.g., their self-efficacy) and thus their behavior (Conway et al., 2004a, b; Hirsch et al., 2004; Marsh & Martin, 2011). That is, a person’s self-concept, heightened via autobiographical memory recall, can therefore elicit a person’s self-efficacy (e.g., I am a diligent researcher who can complete this project; Bandura, 1997; Pillemer & Kuwabara, 2012; van der Bijl & Shortridge-Baggett, 2001). Heightened self-efficacy may, in turn, influence open-ended problem solving (e.g., Brown et al., 2016).

## Experiment 1: Do success autobiographical memories enhance open-ended problem solving?

Experiment 1 aimed to test whether self-efficacy elicited from autobiographical memories helped participants to generate solutions as measured by the MEPS. Since Brown et al. (2016) found that when participants were not limited to recalling related experiences (as in Goddard et al., 2001) and instead could recall any experience where they felt successful and competent, and that this had effect on problem solving, we chose to use their experimental design as a starting point. Experiment 1 therefore tested whether recalling any experiences of success and reflecting on how these experiences reflected strengths also influenced MEPS performance, like in Brown and colleagues’ study. An additional reason was that we could isolate self-efficacy as a predicted mechanism for autobiographical memory helping with open-ended problems. We also opted to follow Brown and colleagues’ approach by using the MEPS (Platt & Spivack, 1975) as the dependent variable, which is the most common and direct way of measuring open-ended problem solving.

Derived data, supplementary material (including task instructions), and pre-registration for Experiments 1 and 2 can be found on the Open Science Framework (https://osf.io/h57ca/). Ethics approval was also granted by the university’s Human Ethics Committee (0000029267) for both experiments outlined in this paper.

## Method

### Participants

Since the effect of autobiographical memory on MEPS in inconclusive, we used G*Power (Faul et al., 2007) to help determine the sample size needed for a medium effect. We therefore pre-registered and recruited a sample size of 200 undergraduate students. Students participated in partial fulfilment of a course requirement. As per preregistered exclusion criteria, the data of 31 participants (15.5% of the data) were removed: seven because two or three of their memories were not specific autobiographical memories, and 21 from the neutral-memory condition because they rated their memories too positive or negative on average (i.e., either below -4 or higher than 4 on the Likert Scale, see below for scale). Data from another three participants were removed because they did not follow the instructions. Therefore, 169 participants were included in analyses: 127 identified as female (75.1%), 36 as male (21.3%), five as another gender (3.0%), and one preferred not to disclose (0.6%). Most participants were 18–24 years of age (142 participants, 84.0%), with the remaining participants under the age of 18 years (14 participants, 8.3%) and between 25 and 34 years (12 participants, 7.1%) and 55 and 64 years (one participant, 0.6%).

### Design and procedure

**Fig. 1:**
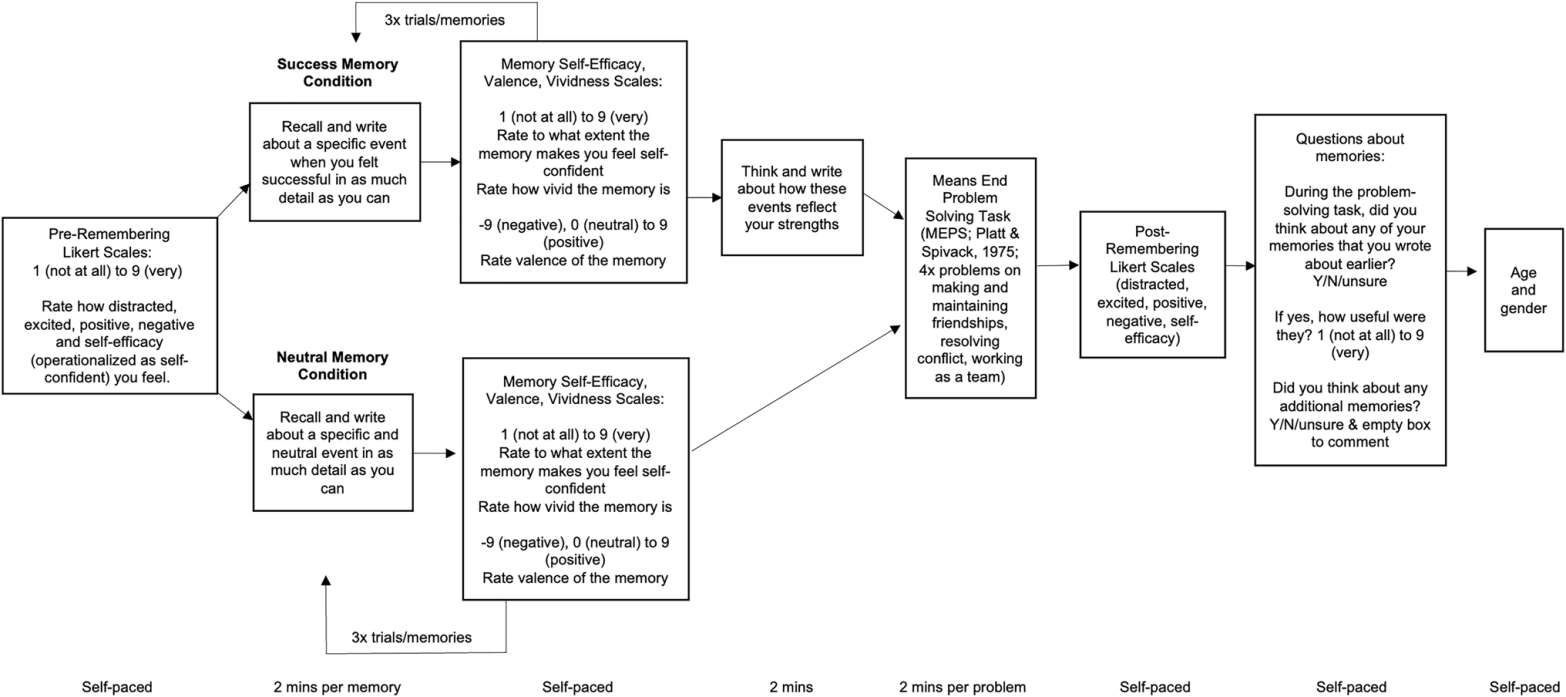
Procedure for Experiment 1

Experiment 1 was a between participant design and was created using the online survey software Qualtrics. Participants used their own computer and completed the experiment at a location and time of their choosing. Participants were randomly assigned to the success-memory condition or the neutral-memory condition and completed the tasks in the order laid out in Fig. 1.

The experiment began with Likert scales which asked participants to rate on a 1 (*not at all*) to 9 (*very*) Likert scale how distracted, excited, positive, negative, and self-efficacious they felt (i.e., *How distracted/excited do you feel? How positive/negative is your mood? How self-confident do you feel right now?*). The purpose of these Likert scales was to ensure that participants felt similarly before commencing the experiment and to capture any change in self-efficacy. Following previous research (Brown et al., 2016; Raeder et al., 2019), we operationalized self-efficacy as how self-confident the participant felt: These constructs are related in that self-efficacy is defined as the confidence in executing a particular task (Bandura, 1997, 2009; van der Bijl & Shortridge-Baggett, 2001). Note that the self-efficacy Likert scale scores before and after the experimental manipulation are referred to as “pre- and post-remembering self-efficacy” (also refer to Fig. 1 for terminology).

Following random assignment to either the success- or neutral-memory condition, participants were assigned to recall and write about either three specific autobiographical memories of when they felt competent and successful (i.e., success-memory condition) or three specific neutral autobiographical memories that did not elicit any strong emotion (i.e., neutral-memory control condition). That is, in the success-memory condition, participants were asked to recall three particular times where they succeeded and felt good about themselves because they did something that proves they can cope with challenges (using the instructions from Brown et al., 2016). Participants in the neutral-memory condition were asked to recall three specific neutral events that do not bring up any strong emotion. As we wanted to heighten self-efficacy in the success-memory condition, we asked participants to recall specific and detailed memories. This is because highly arousing (compared to low arousing) memories tend to be more subjectively vivid (Talarico, et al., 2004) and recalled with more detail (Simpson & Sheldon, 2020). They were given 2 min to recall and write about each memory.

After writing about a memory, participants rated their self-efficacy and the memory’s vividness on a 1 (*not at all*) to 9 (*very*) Likert scale. The self-efficacy question asked: *When you think and write about your memory, to what extent does it make you feel confident in yourself?* On a –9 (*negative*), 0 (*neutral*), and 9 (*positive*) Likert scale, participants reported the memory’s valence. This was to test whether participants in the success-memory condition thought about self-efficacious experiences in comparison to participants in the neutral-memory condition. Although we measured self-efficacy at pre- and post-remembering, any change in self-efficacy could also be influenced by tasks completed after recalling their memory like the MEPS. Therefore, we also measured self-efficacy directly after participants recalled their autobiographical memory. Thus, for each participant, the mean was taken from the self-efficacy Likert scale across the three memories to provide an overall ‘memory self-efficacy’ score. It is important to note that in some research contexts ‘memory self-efficacy’ can mean people’s evaluation of their memory abilities (e.g., Beaudoin & Desrichard, 2011). For this study, we used this term to refer to how self-confident a participant felt immediately after recalling their experiences*.* After recalling their success autobiographical memories, participants in the success-memory condition also thought and wrote about how their memories reflected their strengths.

Participants then completed an adapted version of the MEPS task (Platt & Spivack, 1975). Participants were given open-ended problem scenarios (i.e., resolving conflict, making and maintaining friendships, working as a team), along with their positive endings, and were asked to generate as many solutions as possible that the protagonist can take to move them from the problem to the positive ending.

At the end, participants also answered questions about whether they thought about their memories during the task and how useful they thought their memories were. Since people report that they draw on their autobiographical memories during problem solving (Bluck, et al., 2005; Bluck & Alea, 2011; Webster, 1993, 1997, 2003), we also asked participants if they thought about any additional memories and, if so, what these were. Lastly, we asked for gender and age. Experiment 1 took 30 min to complete and ended with an overview of the research project and optional readings.

### Coding and reliability

To measure problem solving, we counted the number of solutions (also known as ‘relevant means’ in the MEPS task manual; Platt & Spivack, 1975) that helped the protagonist in the problem scenario move towards the positive ending. Solutions across the four problem scenarios were summed to provide an overall ‘problem-solving score’, with higher scores indicating better problem solving. ‘Irrelevant means’ (i.e., an ineffective solution within the context of the problem scenario) and ‘no means’ (i.e., commentary about the task, repetitions, and vague solutions) were also counted, as outlined in the MEPS manual. Irrelevant means and no means were collapsed to create an overall ‘non-solutions score’.

Guided by Syed and Nelson (2015) to establish reliability, one secondary coder coded 20% of the problem-solving answers for solutions and non-solutions. We calculated intraclass correlation coefficient (ICC) estimates and their 95% confidence intervals (CIs) based on a single rating (as only the primary coder scores were used in the final analysis), consistency (as problem-solving scores are continuous), two-way mixed-effects model (as coders were not selected at random; for more detail, see Koo & Li, 2016). There was good, significant reliability for problem-solving scores, ICC = .81, *F*(33, 33) = 9.72, *p* < .001, 95% CI (.67, .90), and non-solutions scores, ICC = .89, *F*(33, 33) = 16.80, *p* < .001, 95% CI (.79, 94). The primary and secondary (i.e., reliability) coders were unaware of the participants’ experimental condition.

### Data normality testing

Data were graphed (i.e., histograms and p-p plots) and analyzed, and tested for skewness and kurtosis (guided by Field, 2017). Considering their non-normal distributions, non-parametric tests were used when assessing distraction, non-solutions, memory valence, memory vividness and memory self-efficacy scores. All other measures were normally distributed.

### Manipulation check

Manipulation checks were run to ensure that participants in the success-memory condition thought about self-efficacious memories, and that these autobiographical memories heightened self-efficacy. Furthermore, we tested whether participants felt similarly before the experiment and therefore randomization had successfully created equivalent groups.

In sum, participants in the success-memory condition increased their self-reported self-efficacy from pre- to post-remembering (*p* = .005), whereas the participants in the neutral-memory condition did not (*p* = 1.00). Participants in the success-memory condition also rated their memories as making them feel more self-confident (*p* < .001; i.e., memory self-efficacy scores) compared to participants in the neutral-memory condition. Thus, the manipulation of success autobiographical memories appeared to have worked. There were also no significant pre-remembering differences in self-efficacy, distracted, excited, negative and positive mood Likert scales, so participants in the two conditions felt similarly before the experiment (all p > .05; see Open Science Framework (https://osf.io/h57ca/) for inferential statistics and Table 1 for descriptive statistics).

**Table 1:** Descriptive statistics for pre- and post-remembering self-efficacy, memory self-efficacy, memory valence, memory vividness, problem-solving, non-solutions, and episodic detail scores by condition

*n*	Success-memory condition		Neutral-memory condition	
	95		74	
	Mean (SD)	Median (IQR)	Mean (SD)	Median (IQR)
Pre-Remembering Self-Efficacy	4.74 (1.91)	5.00 (3.00 – 6.00)	5.00 (1.78)	5.00 (4.00 – 6.00)
Post-Remembering Self-Efficacy	5.38 (1.83)	5.00 (4.50 – 7.00)	4.86 (1.75)	5.00 (4.00 – 6.00)
Memory Self-Efficacy	7.37 (1.10)	7.67 (6.83 – 8.00)	4.78 (1.38)	5.00 (4.00 – 5.67)
Memory Valence	6.21 (2.61)	7.00 (5.00 – 7.67)	1.03 (1.28)	0.83 (0.08 – 2.00)
Memory Vividness	7.11 (1.21)	7.33 (6.67 – 8.00)	6.01 (1.54)	6.00 (5.08 – 7.25)
Problem-Solving Score	14.20 (5.12)	13.00 (10.5 – 17.0)	15.00 (5.69)	15.50 (11.00 – 18.80)
Non-Solutions Score	3.09 (2.59)	2.00 (1.00 - 4.00)	3.66 (3.34)	3.00 (1.00 - 5.00)
Episodic Detail Score	13.20 (5.34)	12.00 (9.33 – 16.80)	16.20 (8.09)	14.80 (9.75 – 21.80)

## Results and discussion

### Success autobiographical memories did not enhance problem solving

Although success autobiographical memories heightened self-ratings of self-efficacy and were rated as more self-efficacious than neutral autobiographical memoires, the main hypothesis that participants in the success-memory condition would generate significantly more solutions to open-ended problems than participants in the neutral-memory condition was not supported. This was because there was no significant difference between the conditions on problem-solving scores, *t*(167) = .94, *p* = .349 (see Table 1 for descriptive statistics). Participants in the success- and neutral-memory conditions also did not differ significantly on non-solutions scores, *U* = 3270, *p* = .434.

### Secondary analysis: Self-efficacy positively correlated with problem solving for participants in success-memory condition

**Fig. 2:**
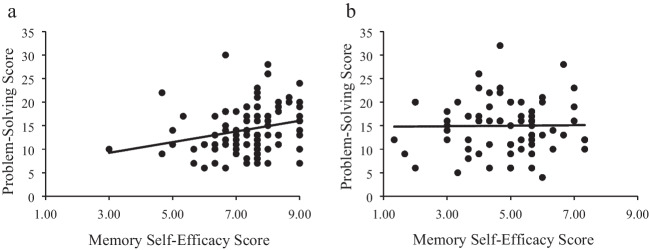
Memory self-efficacy scores by problem-solving scores for the (**a**) success-memory condition and (**b**) neutral-memory condition. *Note.* The line is the linear regression. **Graph a.** success-memory condition, *rs* = .24, *p* = .018. **Graph b.** neutral-memory condition, *r* = .01, *p* = .911

Although we found no experimental effect of success autobiographical memories on problem solving, considering our primary research focus we tested if self-efficacy was associated with problem solving. For participants in the success-memory condition, correlational analyses indicated a positive relationship between memory self-efficacy scores and problem-solving scores (*r*s = .24, *p* = .018). In contrast, this relationship was non-significant for participants in the neutral-memory condition (*r* = .01, *p* = .911; see Fig. 2). For participants in the success-memory condition, problem-solving scores also positively correlated with pre- (*r* = .20, *p* = .047) and post- (*r* = .21, *p* = .041) remembering self-efficacy. Pre- and post-remembering self-efficacy did not correlate with problem solving for participants in neutral-memory condition (both *p* > .05).

Given that participants in the success-memory condition had significantly higher memory self-efficacy scores than participants in the neutral-memory condition, and that self-efficacy scores positively correlated with problem solving, why did we not find a significant experimental effect of success autobiographical memories as hypothesized? Since problem solving positively correlated with pre-remembering self-efficacy, one possibility is that the relationship between self-efficacy and problem solving was due to pre-existing individual differences. Countering this explanation, however, is that these relationships were non-significant for participants in the neutral-memory condition. Therefore, it appears the relationship between self-efficacy and problem solving was unique to the participants in the success-memory condition.

### Exploratory post hoc analyses

Given theory and evidence that recalling memories support problem solving, one explanation for the lack of difference between the success-memory and neutral-memory conditions is that participants were using memories to direct problem solving to a similar extent in both conditions. This might happen if participants in the neutral-memory condition spontaneously recalled additional autobiographical memories while completing the problem-solving task and/or if the neutral memories we asked them to recall supported problem solving regardless of not impacting self-efficacy. Therefore, we conducted non-pre-registered post hoc analyses to explore these two options.

### Additional autobiographical memories were helpful and related to the problem

Given that people report they draw on helpful experiences when faced with a problem (Bluck et al., 2005; Bluck & Alea, 2011; Webster, 1993, 1997, 2003), some participants may have done this in our experiment which could have improved problem-solving scores. Indeed, across the sample (*n* = 169), 72 participants (success-memory condition *n* = 43, neutral-memory condition *n* = 29) said they recalled additional autobiographical memories (i.e., autobiographical memories we did not request), 23 said they did not, and 74 were unsure. Since the ‘no’ category had a low count, it was combined with the ‘unsure’ category (success-memory condition *n* = 52, neutral-memory condition *n* = 45). An independent samples *t*-test revealed that participants who thought about additional memories identified significantly more solutions (*M* = 16.1, *SD* = 5.70) than those in the combined category of those who were unsure and those that did not (*M* = 13.4, *SD* = 4.84), *t*(167) = -3.33, *p* = .001, Cohen’s *d* = .52.

Participants who recalled additional autobiographical memories during the problem-solving task were asked about the content of their additional memories. Their responses were coded for themes, using an inductive thematic analysis, within an essentialist and experiential perspective (guided by Braun & Clarke, 2012, 2022). Responses were first read to become familiar with the data, initial themes were then generated, and then those themes were reviewed and refined. Four main themes emerged: (1) related experiences (95.8% of responses; e.g., “*…I thought about group assignments that I”ve done at school*”, “*I thought about similar experiences I had been through*”); (2) applying their experience (31.9% of responses; e.g., “*I thought about what worked or didn”t work for me*”, “*I applied my experience to the problem*”); (3) specific lesson or solution to apply to the problem (30.6% of responses; “*I remember when I moved into the university hostel and left my door open so people could come talk to me*”); and (4) social modelling (11.1% of responses; “*I drew from my friend‘s experience and thought about what they did to overcome the situation*”). Note that responses could have more than one theme. A secondary coder coded 20% of responses for reliability. There was 100% agreement for three out of the four themes identified. Coders had 92.8% agreement for the second theme – applying their experience – and using Cohen’s *k*, there was almost perfect agreement between coders for this theme, *k* = .85, p < .001 (Viera & Garrett, 2005).

### Were neutral autobiographical memories helpful? The potential role of episodic detail

Another potential reason that we found no significant effect of success autobiographical memories on problem solving is that neutral autobiographical memories were also helpful. We asked for specific and detailed success autobiographical memories because vivid memories may heighten self-efficacy (Holland & Kensinger, 2010). We also asked for specific and detailed neutral autobiographical memories to hold these features constant across the two conditions, but perhaps this could have been helpful. This is because the level of episodic detail (i.e., the event, place, time, perception, emotion, and thought details about an event) in memories people recall has been found to positively correlate with problem solving as measured by the MEPS (Madore & Schacter, 2014; Vandermorris et al., 2013). Experimental evidence also indicates that when people receive coaching to recall a great amount of episodic detail, their problem solving (measured by the MEPS) improves (Jing et al., 2016, 2020; Madore & Schacter, 2014; McFarland et al., 2017). Thus, using the Adapted Autobiographical Interview Scoring Manual (Addis et al., 2008), we coded all memories for episodic detail (see OSF link above for more information on the coding scheme). Reliability for episodic detail was assessed in the same way as the problem-solving coding, which indicated good significant reliability, ICC = .87, *F*(33, 33) = 14.47, *p* < .001, 95% CI (.76, .93).

**Fig. 3:**
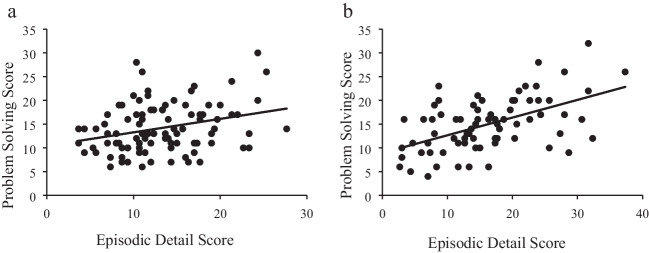
Memory episodic detail scores by problem-solving scores for the (**a**) success-memory condition and (**b**) the neutral-memory condition. *Note.* The line is the linear regression. **Graph a.** success-memory condition, *r* = .29, *p* = .004. **Graph b.** neutral-memory condition, *r* = .53, *p* < .001

Results indicated that neutral autobiographical memories had significantly more episodic detail than success autobiographical memories, *t*(120.25) = 2.77, *p* = .006, *d* = .45 (see Table 1): Note that the *t* statistic and degrees of freedom were adjusted as the assumption of homogeneity of variances was not met with the Levene’s *F* test, *F*(167) = 13.83, *p* < .001. Furthermore, there was a positive relationship between episodic detail and problem-solving scores for participants in both conditions, but this relationship was significantly stronger (${Z}_{Difference}$ = 1.99, *p* = .023) for participants in the neutral-memory condition (*r* = .53, *p* < .001; see Fig. 3) than for participants in the success-memory condition (*r* = .29, *p* = .004).

The neutral experiences provided by participants tended to be recent, concrete, mundane tasks (e.g., making breakfast that morning, going for a walk the day before). Thus, the neutral autobiographical memories’ accessibility and concrete nature may have led to more episodic detail in comparison to success autobiographical memories, which may have helped with the problem-solving task (Schacter & Madore, 2016). We focused on this possibility for Experiment 2. Therefore, we replicated the success- and neutral-memory conditions from Experiment 1 to test whether either or both promoted problem-solving compared to a control condition that did not elicit self-efficacy or episodic detail.

## Experiment 2: Do success and neutral autobiographical memories enhance problem solving?

Experiment 2 tested the possibility that one possible reason that we found no significant difference between conditions in Experiment 1 is that the neutral autobiographical memories acted as a type of specificity induction (for review, see Schacter & Madore, 2016) and were as helpful for problem solving as the success memories. As noted earlier, greater episodic detail can be helpful for problem solving as measured by the MEPS (Schacter & Madore, 2016). Experiment 2 therefore replicated the methodology of Experiment 1, but a control condition (a letter-counting task) was added that neither enhanced self-efficacy nor elicited episodic detail. If detailed neutral autobiographical memories promote problem solving, then both the neutral- and success-memory conditions would differ significantly from the letter-counting condition with respect to the number of solutions generated.

To assess and control for pre-existing individual differences, as well as test for more subtle effects, Experiment 2 asked participants to complete the problem-solving task before the memory and control tasks. Using a mixed design for Experiment 2 also meant we could examine whether self-efficacy and episodic detail were correlated with problem solving assessed before the memory manipulation and thus better understand whether correlations reflect pre-existing individual differences. We predicted that participants in the success- and neutral-memory conditions would demonstrate enhanced problem solving from pre- to post-remembering, but participants in the letter-counting condition would not. We also predicted that participants in the success- and neutral-memory condition would have higher problem-solving scores than the letter-counting condition after the experimental manipulation.

## Method

### Participants

Since we found no effect in Experiment 1, we increased our sample size to one that G*Power (Faul et al., 2007) indicated was sufficient to detect a small to medium effect. Thus, we preregistered a sample size of 420. As in Experiment 1, participants were undergraduate students who participated in partial fulfilment of a course requirement. As per preregistered exclusions, the data of 79 participants (18.8%) were removed because they did not recall specific autobiographical memories (*n* = 6) or they rated the letter-counting task or their neutral autobiographical memories too positively or negatively (i.e., either below -4 or higher than 4 on the Likert Scale; *n* = 53). Data from an additional 20 participants were removed because they did not follow the instructions or complete the experiment. Therefore, 341 participants were included in the analyses: 235 identified as female (69.1%), 93 as male (27.4%), two as another gender (0.6%), ten preferred not to disclose (2.9%), and one person did not answer. Most of the sample was 18–24 years of age (301 participants, 88.5%), with the remaining participants younger than the age of 18 years (12 participants, 3.5%), 25–34 years (23 participants, 6.8%), 45–54 years (two participants, 0.6%), and 55–64 years (two participants, 0.6%).

### Design and procedure

Experiment 2 followed the same procedure as Experiment 1, except the participants completed the MEPS task before and after the experimental manipulation and we added a letter-counting condition. That is, after completing the pre-remembering/task Likert scales, participants completed two problem scenarios selected at random. Participants were then randomly assigned to either the success-memory, neutral-memory, or letter-counting condition, and then completed the tasks in the same order as Experiment 1. The letter-counting task was chosen because it was unlikely to elicit self-efficacy or strong emotion and did not require autobiographical memory retrieval. The letter-counting task is similar to the letter cancellation task (i.e., crossing out the target letter with a pen) which requires attention and visual scanning (Deng et al., 2019; Uttl & Pilkenton-Taylor, 2001). Like the memory conditions, there were three trials for the letter-counting condition, with a different letter for each trial. Participants had 2 minutes per trial and then indicated how self-confident they felt after the task (i.e., their self-efficacy) and the valence of the task.

### Coding and reliability

Following the same procedure as Experiment 1, problem-solving answers and memories were coded for (non) solutions and episodic detail, respectively, and reliability was assessed. Reliability analysis indicated there was excellent, significant reliability for episodic detail, ICC = .91, *F*(48, 48) = 20.90, *p* < .001, 95% CI (.84, .95), and problem-solving scores, ICC = .91, *F*(67, 67) = 20.57, *p* < .001, 95% CI (.85, .94), and good, significant reliability for non-solution scores ICC = .77, *F*(67, 67) = 7.57, *p* < .001, 95% CI (.65, .85).

### Data normality testing

Normality testing was conducted in the same way as Experiment 1, which indicated that the distributions of excited, distracted, memory valence, memory self-efficacy, memory vividness, episodic detail, problem-solving score and non-solutions score were non-normal in one or more conditions. Thus, we used non-parametric or robust tests (WRS2 package in R) when analyzing these variables. All other measures were normally distributed.

### Manipulation check

The same manipulation checks were conducted as in Experiment 1 (see OSF link above for all inferential and descriptive statistics). Following pre-registered exclusion criteria of participants who rated their neutral memories or the letter-counting task too positively or negatively (above 4 or below -4 on the Likert scale), participants in the letter-counting condition were significantly less positive compared to participants in success- and neutral-memory conditions before starting the experiment (both *p* < .05). Participants in the letter-counting were also significantly less excited compared to participants in the success-memory condition before starting the experiment (*p* = .020). That is, after exclusions, participants in the letter-counting condition were significantly less positive and excited compared to the memory conditions. There were no significant differences between pre-remembering/task distractedness and negative scales (both *p* < .05), however.

Importantly, there were no significant differences in pre-remembering/task self-efficacy scores between conditions (all *p* = 1.00). Self-efficacy scores of participants in the success-memory condition also increased from pre- to post-remembering (*p* < .001), but scores in the neutral-memory and letter-counting conditions did not (both *p* = 1.00). Thus, although there were positive and excitement mood differences before starting the experiment, Experiment 2 found that success autobiographical memories still enhanced self-efficacy. Also, the self-efficacy manipulation check results held when exclusions were retained. Additionally, retaining all data did not change the results presented below unless clearly specified and thus exclusions were kept as they were preregistered.

## Results and discussion

### Success or neutral autobiographical memories did not enhance problem solving

Although participants in the success-memory condition showed increases in self-efficacy, as well as had higher memory self-efficacy scores, as for Experiment 1, there was no significant experimental effect of success autobiographical memories on problem solving. Neutral autobiographical memories were also recalled with more episodic detail than success autobiographical memories and episodic detail was positively correlated with post-remembering problem solving (see below for results), yet neutral memories too produced no experimental effect on problem solving. Indeed, a 2 (timepoint: pre- and post-remembering/task) × 3 (condition: success, neutral, letter-counting) robust repeated-measures ANOVA using 20% trimmed means revealed no significant main effect of timepoint, *F*(1, 182.47) = 0.65, *p* = .420, condition, *F*(2, 122.59) = 0.70, *p* = .498, nor interaction, *F*(2, 122.75) = 0.63, *p* = .534 (Table 2).

**Table 2:** Descriptive statistics for pre- and post-remembering/task and total problem-solving scores, non-solution scores, self-efficacy scores

	Success-Memory Condition	Neutral-Memory Condition	Letter-Counting Condition
*n*	126	118	97
Pre-Remembering/Task Problem-Solving Score*	7.26 (0.30)	7.18 (0.26)	6.88 (0.38)
Post-Remembering/Task Problem-Solving Score*	7.37 (0.35)	7.64 (0.36)	6.93 (0.35)
Pre-Remembering/Task Non-solution Score*	1.34 (0.17)	1.24 (0.17)	1.66 (0.19)
Post-Remembering/Task Non-Solution Score*	1.04 (0.17)	1.14 (0.13)	1.64 (0.20)
Total Problem-Solving Score*	14.96 (0.63)	14.83 (0.59)	13.78 (0.65)
Total Non-solutions Score*	2.63 (0.22)	2.43 (0.19)	3.36 (0.27)
Pre-Remembering/Task Self-Efficacy	4.87 (2.05)	4.81 (2.02)	4.66 (2.05)
Post-Remembering/Task Self-Efficacy	5.72 (1.99)	4.91 (1.94)	4.92 (1.75)

*Note*. * Denotes trimmed mean (20%) and the standard error of the trimmed means, pre- and post-remembering/task self-efficacy are arithmetic mean and standard deviation

For non-solutions, there was a main effect of condition, *F*(2, 121.54) = 3.33, *p* = .039. Robust Independent Samples T-tests revealed that the participants in the letter-counting condition reported more total non-solutions than participants in the success-, *t*(121) = 2.07, *p* = .04, and neutral-, *t*(109) = 2.81, *p* = .006, memory conditions. Participants in the success- and neutral-memory conditions did not differ on total non-solutions scores, *t*(143) = .68, *p* = .495. The difference between participants in the letter-counting and memory conditions on non-solutions is likely due to participant exclusion criteria differentially affecting the letter-counting condition, however. This is because when all data were retained the main effect was no longer significant (*p* = .090). After exclusions, excitement and positive mood were significantly lower in the letter-counting condition before completing the problem-solving task compared to the memory conditions. Lower mood may therefore influence the number of non-solutions generated. This difference in mood likely occurred because we excluded participants who rated the letter-counting task too positively.

### Memory self-efficacy scores positively correlated with problem solving for participants in success-memory condition

As per Experiment 1, we wanted to see if self-efficacy positively correlated with problem solving. For participants in the success-memory condition, memory self-efficacy scores positively correlated with pre- (*r*s = .29, *p* < .001) and post- (*r*s = .24,* p* = .011) remembering problem-solving scores. Memory/task self-efficacy scores did not correlate with either pre- or post-remembering/task problem-solving scores for participants in the neutral-memory condition (pre-remembering *r*s = .16, *p* = .090; post-remembering *r*s = .17, *p* = .065) or in the letter-counting condition (pre-task *r*s = -.18, *p* = .072; post-task *r*s = .010, *p* = .919). Pre- or post-remembering/task self-efficacy did not correlate with pre- or post-remembering/task problem-solving for any conditions (all *p* > .05). Although memory self-efficacy positively correlated with pre-remembering problem-solving scores, this relationship was again unique to participants in the success-memory condition. Yet we observed no experimental effect of success autobiographical memories.

### Episodic detail positively correlated with post-remembering (not pre-remembering) problem solving

Following Experiment 1 findings, we also wanted to investigate whether neutral autobiographical memories were recalled in more detail and whether episodic detail correlated significantly with problem solving. As expected, participants in the neutral-memory condition recalled significantly more episodic detail (*Mdn* = 15.2, *IQR* = 10.7 - 20.0) compared to participants in the success- memory condition (*Mdn* = 9.5, *IQR* = 6.3 - 14.0; *U* = 4111, *p* < .001).

Additionally, the number of episodic details recalled by participants in the neutral-memory condition positively correlated with their post-remembering problem-solving scores (*r* = .27, *p* = .003), but not their pre-remembering problem-solving scores (*r* = .14, *p* = .129). The same pattern of results was evident for participants in the success-memory condition: episodic detail significantly correlated with post-remembering problem-solving scores (*r*s = .31, *p* < .001) but not with pre-remembering problem-solving scores (*r*s = .16, *p* = .083). In sum, there was a positive correlation between episodic detail and problem solving but only after (not before) recalling neutral and success autobiographical memories. This suggests that this relationship is not due to pre-existing individual differences. Yet we observed no experimental effect of autobiographical memories.

### Additional, related autobiographical memories were helpful

Following the approach taken in Experiment 1, we asked participants whether they recalled additional autobiographical memories (or any autobiographical memories for the letter-counting condition) that were not specifically requested for during the problem-solving task (either before or after remembering or the letter-counting task). From the sample (*n* = 341), 198 participants (success-memory condition *n* = 68; neutral-memory condition *n* = 67; letter counting condition *n* = 63) said they recalled additional autobiographical memories, whereas 102 said they did not, 40 said they were unsure, and one participant did not select an answer. Since the count for the ‘unsure’ category was low, it was combined with the ‘no’ category (success-memory condition *n* = 57; neutral-memory condition *n* = 51; letter-counting condition *n* = 34). Like in Experiment 1, participants who thought about additional autobiographical memories identified significantly more total solutions (*M* = 15.6, *SD* = 5.97) than the combined category of those who did not and who were unsure (*M* = 14.3, *SD* = 6.17), *t*(337) = -2.00, *p* = .049, Cohen’s *d* = 0.22.

We investigated whether the four themes from Experiment 1 also emerged from the data in Experiment 2. Responses revealed the same themes: (1) related experiences (83.3% of responses); (2) applying their experience (24.2% of responses); (3) specific lesson or solution to apply to the problem (21.2% of responses); and (4) social modelling (6.1% of responses). A secondary coder coded 20% of the responses for reliability of the themes. Reliability analyses indicated substantial reliability when calculating Cohen’s kappa (Viera & Garrett, 2005): (1) 96% agreement, *k* = .66, *p* < .001; (2) 85% agreement, *k* = .60, *p* < .001; (3) 92.6% agreement, *k* = .76, *p* < .001; (4) 97.1% agreement, *k* = .76, *p* < .001.

In sum, some participants recalled additional (or any for the letter-counting condition) experiences, and this approach was associated with better problem solving. The most common theme, as in Experiment 1, was recalling related experiences.

## General discussion

Taking an experimental approach, the overarching research goal was to investigate the directive function of autobiographical memory; that is, the conditions under which autobiographical memory guides and directs a person during open-ended problem solving. Our research therefore had two aims: to experimentally test for the directive function as experimental evidence is limited and results are mixed (Beike et al., 2020; Pillemer & Kuwabara, 2012), and to test whether self-efficacy elicited from autobiographical memory recall is an important factor that supports open-ended problem solving (Bandura, 1997; Brown et al., 2012; Brown et al., 2016; D’Zurilla et al., 2004). We therefore experimentally tested whether success autobiographical memories – any experience when the participant felt successful and competent – were helpful for generating solutions to open-ended problem scenarios.

Across two experiments, success autobiographical memories had no significant effect on open-ended problem solving (as measured by MEPS task; Platt & Spivack, 1975), however. That is, undergraduate students who recalled times when they felt successful and competent did not generate significantly more solutions to open-ended problems from pre- to post-remembering or compared to participants who recalled neutral autobiographical memories, or who completed a neutral letter-counting task.

We did find, however, that across both experiments, for participants who recalled success autobiographical memories, self-efficacy after recalling their success experience was positively correlated with their problem solving. This relationship was non-significant for participants who recalled neutral autobiographical memories or completed a letter-counting task before problem solving. Constraining our interpretation of this finding, however, is that self-efficacy was correlated with problem solving before remembering in Experiment 2. This correlation may therefore not be driven by a causal link from success memories to problem solving, but rather due to pre-existing individual differences.

Self-efficacy in this study was measured by how self-confident a person felt. This was to follow researchers in the area (Brown et al., 2016; Raeder et al., 2019), particularly Brown and colleagues, whose procedure we adapted for our research. Yet, self-confidence and self-efficacy are different, albeit related, constructs (Bandura, 1997; van der Bijl & Shortridge-Baggett, 2001). Feelings of self-confidence may influence a person’s self-efficacy – their belief in executing a task – but self-efficacy typically refers to a person’s belief in their ability to undertake a specific task (Bandura, 1997; van der Bijl & Shortridge-Baggett, 2001). Future research could therefore include a precise measure of self-efficacy. For instance, self-efficacy could measure a person’s belief they can overcome the problem described in the problem-solving task, or how easy they found the problem-solving task, or more directly ask whether they believe their ability demonstrated in their success memory translates to their ability to complete the current problem-solving task (Bandura, 2009; van der Bijl & Shortridge-Baggett, 2001).

### Why no effect of success autobiographical memories?

Putting aside measurement limitations, across both experiments, participants who recalled success autobiographical memories reported enhanced self-efficacy, and self-efficacy was positively correlated with their problem solving. Yet success autobiographical memories had no effect on open-ended problem solving. Why, therefore, did we find no significant experimental effect of success autobiographical memories? We have suggested one possibility is that these relationships are due to pre-existing individual differences. That is, people who are generally better problem solvers are also generally higher in self-efficacy. Our data and other research do suggest other possibilities, however.

#### Spontaneous recall of helpful autobiographical memories

One possibility is that some participants across the conditions recalled other helpful autobiographical memories. Across both experiments, some participants recalled autobiographical memories that we did not specifically ask for, and these participants generated significantly more solutions than those that did not. Thematic analyses revealed that most of these unrequested memories were experiences that resembled the problem scenario. Although recalling related experiences seems like a logical strategy to improve problem solving and more akin to how the directive function is typically defined, surprisingly no experimental research suggests that this is helpful. For instance, Goddard et al. (2001) found that asking participants to recall autobiographical memories that the MEPS problem scenarios reminded them of had no effect on their subsequent problem solving.

Furthermore, if these additional, unrequested, related autobiographical memories are helpful for problem solving, this would suggest that some people spontaneously recall helpful autobiographical memories so any experimental manipulation may fail to differentiate conditions. Therefore, experimental effects may depend on the sample, such that autobiographical memory recall experimental manipulations may only benefit people who do not typically use their autobiographical memories to solve problems.

#### Sample and experimental design factors

Indeed, a major difference between this study and that of Brown et al. (2016) is sample characteristics. Brown et al. found an effect of success autobiographical memories on open-ended problem solving (as measured by the MEPS) and our experiments are based on their approach. Their sample, however, was American combat veterans, 90% male, and between 20 and 60 years of age, whereas the current sample was New Zealand undergraduate students, of whom the majority identified as female and were between the ages of 18 and 24 years. Differences in psychopathology, gender, and age could explain the difference in findings between the studies. For instance, while Brown and colleagues found an effect of success autobiographical memories on problem solving regardless of PTSD status, combat veterans are likely to have more psychological difficulties than students (Ganly et al., 2017; Hoge et al., 2006). Psychological difficulties, particularly depression, have been associated with lower levels of open-ended problem-solving ability (Williams et al., 2007). Women also tend to elaborate more when they recall personal memories (Fivush, 2011) and younger people report that they use their memory in more a directive way compared to older people (Bluck & Alea, 2009). Recalling success autobiographical memories may have therefore benefited Brown and colleagues’ sample more so than our own because our sample were already good problem solvers who spontaneously used their memories.

Other experimental studies have also found an effect of self-efficacious autobiographical memories on exercising (Biondolillo & Pillemer, 2015), public speaking (Pezdek & Salim, 2011), and overcoming fear of heights (Raeder et al., 2019). These experimental findings raise the possibility that when a problem or challenge requires a specific behavior, a related and self-efficacious autobiographical memory may help a person enact that specific behavior. Our own findings, and the mixed findings of the limited number of other studies (e.g., Beaman et al., 2007; Goddard et al., 2001), suggest that it is unclear if and how autobiographical memory can help with generating solutions to open-ended problems as measured by the MEPS, however. There are many elements to open-ended problem solving though, in addition to generating solutions to a problem (D’Zurilla et al., 2004). For instance, one must also be able to define the problem, evaluate options, pick the best course of action, and implement and evaluate a solution (D’Zurilla et al., 2004). The effect of autobiographical memory may therefore differ across these different open-ended problem-solving steps and the MEPS may therefore not fully capture the effect of the directive function.

#### The role of episodic detail

Lastly, episodic detail (i.e., the event, time, place, perceptual, emotion, and thought details remembered about an event) positively correlated with problem solving, and more so for neutral autobiographical memories. Therefore, for Experiment 2 we hypothesized that neutral autobiographical memories may have been helpful for problem solving. This is because, in past research, participants who were coached to recall a great amount of episodic detail from a neutral video clip enhanced their subsequent problem solving as measured by the MEPS (for review, see Schacter & Madore, 2016). Schacter and Madore posited that episodic detail helps a person to assemble and maintain a coherent mental scene (i.e., simulating an event or scene in one’s mind), which assists with cognitive tasks like recalling a specific, detailed memory and open-ended problem solving. Thus, if the same cognitive and neural processes are used to recall an autobiographical memory and generate solutions to problems, then perhaps they influence each other (Schacter & Madore, 2016).

Thus, for Experiment 2 we compared neutral and success autobiographical memories to a letter-counting task. Despite neutral autobiographical memories being recalled with significantly more episodic detail, we also found no effect of these types of memories on problem solving. We did find in Experiment 2, however, that episodic detail positively correlated with *post*-remembering problem-solving scores, but not with *pre*-remembering problem-solving scores for both success- and neutral-memory conditions. This may suggest that detailed autobiographical memories may influence subsequent problem solving, but the experimental effect of detailed memories may be hidden due to extraneous factors, like spontaneous recall of helpful memories or sample and experimental design differences as highlighted above.

### Implications for the directive function

What do our findings mean for the directive function? Are autobiographical memories helpful for problem solving, particularly for generating solutions to open-ended problems? Is self-efficacy an important factor to consider? Although the directive function of autobiographical memory seems intuitive – we use our experiences to guide behavior during problems – it appears that the directive function is nuanced and depends on several factors. We found that self-efficacy was positively correlated to problem solving in both experiments. Our findings and others (Biondolillo & Pillemer, 2015; Pezdek & Salim, 2011; Raeder et al., 2019) show that self-efficacy is an important factor to consider when defining and testing the directive function of autobiographical memory. This aligns with how the self and the directive autobiographical memory functions are found to be linked (e.g., Harris et al., 2014). Although we make the point that self-efficacy is important, so too is how a person uses their experience to navigate a current problem (Bluck & Alea, 2011; Bluck et al., 2005; Harris et al., 2014; Pillemer, 2001, 2003, 2009; Webster, 1997, 2003). Indeed, our findings show that those who spontaneously recalled additional, unrequested autobiographical memories related to the problem scenario did better at problem solving than those that did not.

Our findings also show that many factors may influence open-ended problem solving, which makes it difficult to experimentally test. Thus, research on the directive function may benefit from researchers considering what types of memories are most beneficial, and for what types of problems. Also, to consider how a person should recall their experience (e.g., specific, and detailed) and what they should focus on from their experience (e.g., the lessons they learnt and/or how self-efficacious they are). Lastly, researchers may benefit from considering who benefits the most from recalling autobiographical memories during problem solving. Our general discussion has attempted to begin to answer these questions and highlight the complexity of how autobiographical memory directs behavior.

## Open practices statement

Derived data, supplementary material (including task instructions), and pre-registration for both Experiment 1 and Experiment 2 can be found on the Open Science Framework (https://osf.io/h57ca/).

## Data Availability

The datasets generated during and analysed during the current study are available in the OSF repository link provided.
